# Synthesis and characterization of a nanostructure conductive copolymer based on polyaniline and polylactic acid as an effective substrate in proteins impedimetric biosensing

**DOI:** 10.1039/d4ra01061b

**Published:** 2024-04-18

**Authors:** Ehsan Dinpanah, Moslem Mansour Lakouraj, Ebrahim Fooladi, Vahid Hasantabar

**Affiliations:** a Department of Organic-Polymer Chemistry, Polymer Chemistry Laboratory, Faculty of Chemistry, University of Mazandaran Babolsar 47416 Iran lakouraj@umz.ac.ir; b Department of Food Safety and Quality Control, Research Institute of Food Science and Technology (RIFST) Mashhad Iran e.fooladi@rifst.ac.ir

## Abstract

Despite of all the developments in DNA microarray technology, there is not sufficient knowledge about protein abundance or their function in processes such as proteolysis, phosphorylation. Therefore, there is a significant need for direct detection and quantification of proteins, especially in processes such as proteomics, drug design and disease prediction. The present work introduce the new generation of polymeric substrate based on polyaniline and, polylactic acid, which it was used for impedimetric sensor in detection of proteins in particular for bovine serum albumin (BSA). In this copolymerization, the polylactic acid-*block*-polyaniline copolymer (PLA-*b*-PANI) was synthesized to attach polylactic acid and polyaniline using epichlorohydrin as a coupling agent. The structure of synthesized compounds in all steps, were confirmed by FT-IR and, ^1^H-NMR. The thermal properties and, morphology were analyzed by DSC, TGA, and, SEM. Also the electrochemical characteristics of fabricated PLA-*b*-PANI electrode were investigated by Electrochemical Impedance Spectroscopy (EIS) and Cyclic Voltammetry (CV). The results demonstrated that morphology of the PLA-*b*-PANI is sphere shape nanoparticles with dimension less than 100 nanometer diameters and, reasonable thermal properties. PLA-*b*-PANI was used to modify a screen-printed carbon electrode (SPCE) to fabricate a BSA impedimetric sensor. In order to increase the performance of the proposed impedimetric sensor, optimization of incubation time, pH and amount of PLA-*b*-PANI were investigated. The results show that the impedimetric sensor has the highest response when the electrode surface is covered with 5 microliters of PLA-*b*-PANI, and is incubated in BSA solution with pH 6.5 for 5 min. Impedimetric results showed that the PLA-*b*-PANI has excellent properties in reducing the charge transfer resistance and increasing the electron charge transfer rate. The final impedimetric sensor exhibited good repeatability, reproducibility, and chemical stability within the linear concentration range of 0.1–20 μg L^−1^ of BSA, and a detection limit of 0.05 μg L^−1^.

## Abstract 2

Despite of all the developments in DNA microarray technology, there is not sufficient knowledge about protein abundance or their function in processes such as proteolysis, phosphorylation.

## Graphical abstract

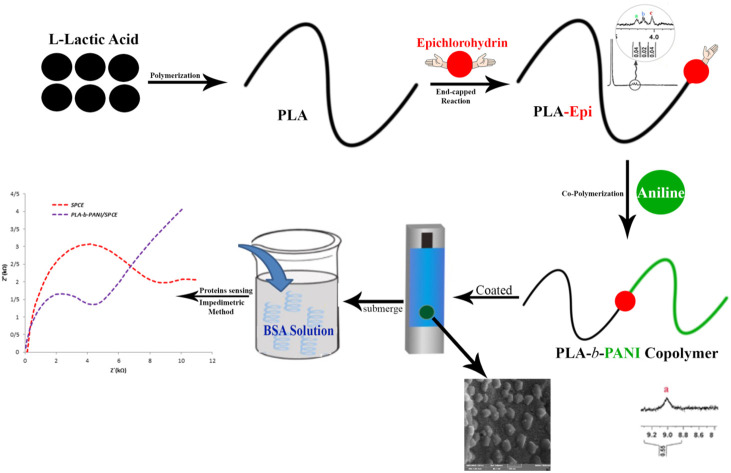

## 1. Introduction

Proteins are an essential part of organisms and play a significant role in metabolic activities, whose behavior can be considered in the manufacture of drugs and diagnosis of diseases because it leads to the reduction of pain and costs associated with it.^1^ Although, there are currently various diagnostic tools available in the commercial markets, many of them are ineffective and, some are very expensive. Therefore, many efforts continue to develop and improve analytical techniques for rapid, reliable and, low-cost detection of a wide range of biological molecules. One of these technologies is the manufacturing and design of nanosensors, which introduce a new generation of sensors with high selectivity and sensitivity, has produced.

Many types of nanoparticles such as metal nanoparticles, oxide nanoparticles, conductive polymers have been used to develop nanosensors and, these nanoparticles play prominent roles in different sensor systems. Among these, conductive polymers have been specially considered in the construction of electrochemical nanosensors.^2^ Conductive polymers (CPs) have attracted particular interest for researchers in various field of high-performance materials.^3–5^ In recent years, many types of nanosensors have been designed using CPs for biological applications. Since the oxidation level of conductive polymers is easily changed by oxidation–reduction mechanisms and, the charge transfer properties of these materials are also influenced by structural parameters, such as diameter and, their dimensions The CPs can provide sensitive and fast responses to specific biological and chemical species. The most widely used CPs in biosensors are polypyrrole, polyaniline, and poly(3,4-ethylenedioxythiophene).^6^

Among these different CPs, polyaniline (PANI) has been widely studied due to its low-cost synthesis, high environmental stability, and good electrical activity. However, the rigidity of the molecular backbone of PANI, which has a highly conjugated π-electron system, leads to its low solubility and poor processability.^7^ Utilization of copolymerization, blending of PANI with other polymers, is the most common method, that have been reported in literature for improving PANI's limitations.^8^ One of the polymers, that are suitable for forming PANI composite, is polylactic acid (PLA). Due to its good mechanical properties and degradability, PLA is used as an attractive substrate for the preparation of electrodes, supercapacitors, immobilization of biomolecules, and static-free packaging.^9^

It has also been reported that the electroactive and biodegradable PLA/PANI composite has been used to make hydrogels, scaffolds, and nanofibers.^10–12^ Along with all the benefits, PLA with high molecular weight is brittleness and has insulating properties. Moreover, the synthesis of PLA-*b*-PANI with a regular structure is practically complex. Therefore, researchers have used PLA and PANI polymer blend, to make flexible electrodes with energy storage capability in the previous.^13,14^ Unlike high molar mass PLA, the preparation of a low molecular weight copolymer of PLA and PANI provides a substrate with excellent flexibility, electrical properties and amphiphilicity, which leads to the reduction of e-waste generation.^15,16^

Biomolecules such as protein and cells can attach to the surface of amphiphilic block copolymer, and due to the ability of the amphiphilic copolymer to form micelles, they can be used in the construction of biosensors.^17^ Nowadays, for the development of new biosensors, many studies have been conducted on the interaction of proteins with polymer surfaces. One of these proteins is BSA, which is commonly used as a model protein for electrochemical sensor studies.^18^ In addition, parameters such as pH, protein concentration, thickness and temperature of the system affect the interaction between proteins and surfaces, so their investigation is important in the design of the biosensor investigated by the researchers. There are several techniques to study the modified electrode surface. Through various methods, EIS is a prominent technique for exploring and characterizing changes in capacitance or charge transfer resistance associated with protein adsorption on a modified electrode surface. Due to high sensitivity, fast response and non-destructive, EIS is considered as a suitable method for label-free biomolecule detection.^19,20^

In this work, we try to design the new modified electrode with block copolymer of PLA and PANI as the novel substrate for preparation of protein detection nano-biosensor. In this regard, first of all, the aniline-end-capped-polylactic acid (PLA-aniline) was synthesized. Then the sequential copolymerization of PLA-aniline with aniline was followed through oxidative radical polymerization to achieve the PLA-*b*-PANI the nano-sized range. The obtained copolymer was subjected to thermal and morphological analysis and the change of its electrochemical properties as a result of interaction with BSA was investigated. Based on the results, it was proved that the synthesized copolymer is a suitable candidate for protein adsorption. Therefore, it can be used as an interactive surface for impedimetric sensors.

## 2. Experimental section

### 2.1. Materials

BSA protein was obtained from Sigma-Aldrich Co. BSA solution (1 mg mL^−1^) was prepared by dissolving BSA in distilled water. KCl, Na_2_HPO_4_·5H_2_O, NaH_2_PO_4_, K_3_ [Fe(CN)_6_] and K_4_ [Fe(CN)_6_] (Merck Co.) used to prepare buffer and probe solutions. Aniline (Merck Co.) was purified by double vacuum distillation. All solvents, ammonium persulfate (APS), epichlorohydrin, sodium ethoxide, Tin (Sn), Na_3_PO_4_·12H_2_O, and hydrochloric acid (HCl) were purchased from Merck Company and used without further purification. l-lactic acid was supplied by Fluka Company.

### 2.2. Synthesis of the PLA by the direct polycondensation

**Scheme 1:**
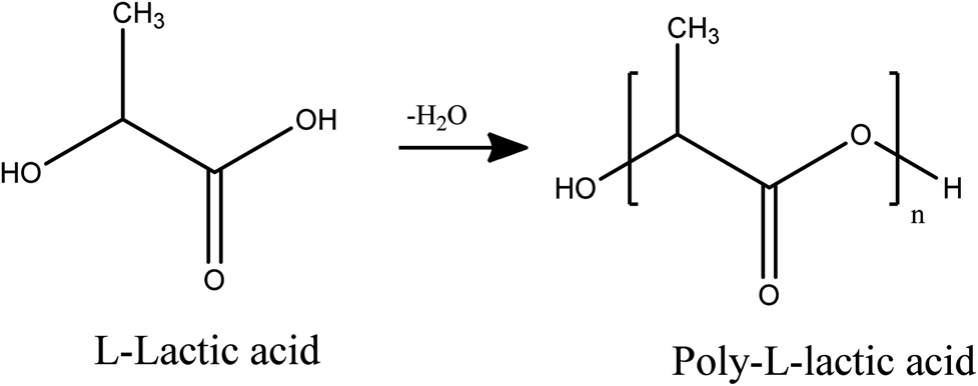
Synthesis of polylactic acid.

The polymerization was carried out according to the modified already reported procedure.^15^ First, 10 g l-lactic acid 90%dissolve in 100 mL chloroform, then added 0.1 g of ground Sn to the resulting solution and separated its water for 2 h at 80 °C by azeotropic method using Dean–Stark. After the end of the first stage of dehydration, 85 mL anisole added to solution and azeotropic dehydration was continued with Soxhlet extraction for 72 h at 180 °C. After the dehydration stage was completed, the reaction mixture was concentrated to about half-volume. The resulting mixture was poured into 350 mL of diethyl ether. Finally, the precipitated crystals were collected by filtration, washed with a large volume of methanol and water, and dried under reduced pressure (Scheme 1).

### 2.3. Synthesis of PLA-*b*-PANI copolymer

**Scheme 2:**
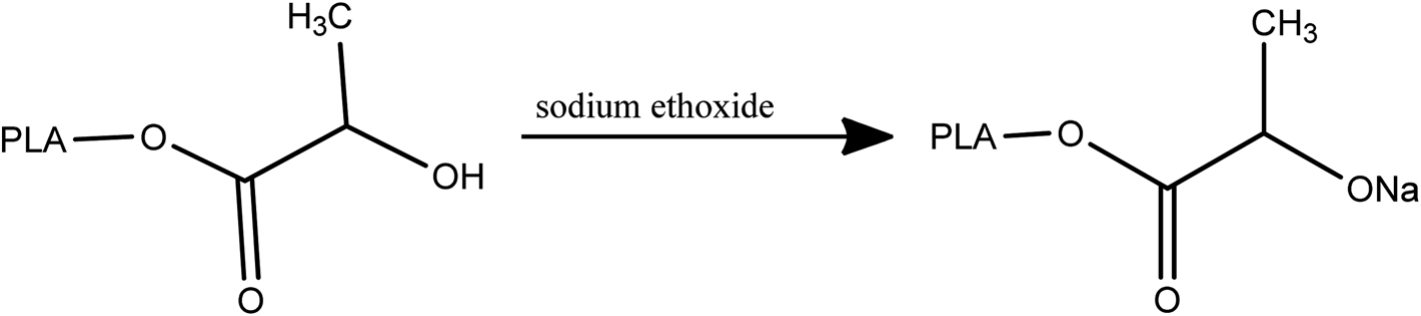
Reaction between PLA and sodium ethoxide.

To synthesis of PLA-*b*-PANIto solution of synthesized PLA (0.05 mg mL^−1^ in DMF), 0.1 mL of sodium ethoxide was added and stirred for 120 min at room temperature (Scheme 2).

**Scheme 3:**
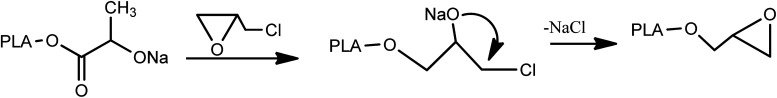
Synthesis of epichlorohydrin-end-capped-polylactic acid (PLA-Epi).

Then, 0.5 mL epichlorohydrin was added to the reaction mixture and was stirred for over 48 h (Scheme 3).

**Scheme 4:**
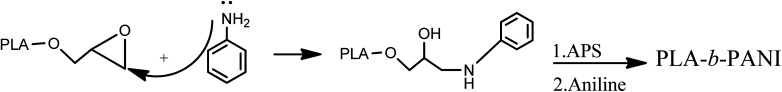
Synthesis of PLA-*b*-PANI.

The resulting mixture was poured into 100 mL of diethyl ether. The obtained precipitated were filtrated and washed with methanol and water. After that, aniline (0.5 mL) and HCl (37%) (0.05 mL) were added to the mixture and stirred for 48 h. In the next step, ammonium persulfate (0.001 mol) was added to mixture and stirred for 60 min (Scheme 4).

After that, the synthesized copolymer was dissolved in hot ethanol and centrifuged to separate the insoluble homopolymer of PANI. The chemical structure of the copolymer was recognized by NMR spectroscopy.^21^

^1^H-NMR (400 MHz, CDCl_3_): 1.245, 1.461, 1.479, 5.201, 5.218, 4.031, 4.145, 4.224, 7.053, 7.099, 7.227, 7.318, 9.015 ppm.

## 3. Electrochemical measurement

All electrochemical experimental measurements were carried out using an Auto lab potentiostat equipped with a three-electrode system consisting of PLA-*b*-PANI/SPCEs as a working electrode, Ag/AgCl and a Pt wire as reference and auxiliary electrodes, respectively. The EIS measurements were performed over a frequency of 0.01 to 100 000 Hz, where OCP potential 154 mV was applied. The Nyquist plots were modeled according to the simplified Randles circulate using Nova version 10 Autolab software. The PLA-*b*-PANI/SPCE electrodes were prepared by drop coating solution containing PLA-*b*-PANI (2 mg mL^−1^ in Chloroform). For SPCE modification, 5 μL of PLA-*b*-PANI solution was placed on electrode and left for 1 h at room temperature. The PLA-*b*-PANI/SPCE is placed in buffer solution (50 mM PBS at pH = 6.5) containing different concentrations of BSA. The EIS measurement of BSA adsorbed on PLA-*b*-PANI/SPCE was performed in solution containing of Fe(CN)_6_^4−^ (5 mM), Fe(CN)_6_^3−^ (5 mM), and KCl (0.1 M).

### 3.1. Characterization

The NMR spectroscopy of all steps was done by Bruker 400 MHz in dimethyl sulfoxide (DMSO-d_6_) and CDCl_3_. Thermal analysis (TGA and DSC) was executed using a TA (Q600) instrument operating at a heating rate of 10 °C min^−1^, from room temperature to 600 °C under an N_2_ atmosphere. Also scanning electron microscopy (SEM) images of sample have been taken by MIRA3 TESCAN. Moreover, the standard four-point probe (Azar Electrode Company, Tabriz, Iran) method was applied to measure the conductivity at room temperature. The molecular mass was determined by Agilent-1100 series in THF at room temperature with flow rate 0.5 mL min^−1^.

## 4. Results and discussion

**Scheme 5:**
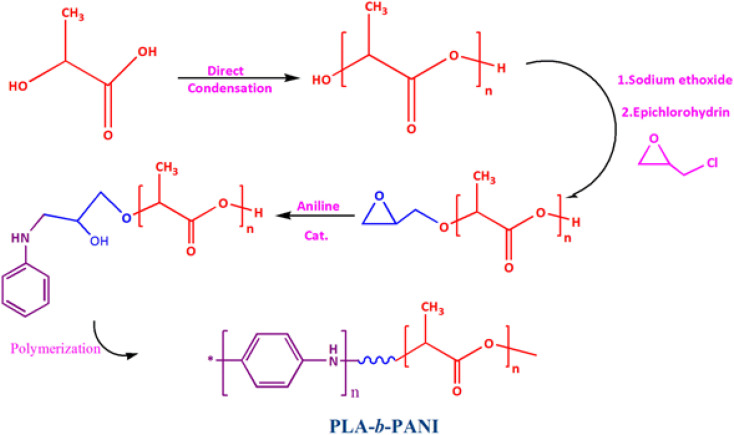
Synthetic pathway for the PLA-*b*-PANI.

Nowadays, investigating the interaction of proteins with other molecular surfaces and their adsorption capacity is one of the most fascinating topics that has attracted the attention of scientists.^22^ In this work, nano PLA-*b*-PANI co-polymer was synthesized in four steps. Scheme 5 summarizes the synthetic pathways for preparation of the PLA-*b*-PANI. Chemical structures, thermal and morphology of nano copolymer were confirmed with FT-IR, NMR, DSC, TGA, and SEM. The interaction of the BSA with nano PLA-*b*-PANI was studied to investigate this potential in impedimetric detection systems in biosensor development. As the results demonstrated, due to the excellent interaction of PANI section with other biologically active compounds and its hydrophobic property, as well as owing to processability of PLA copolymer, the prepared compound could be the suitable candidate for manufacturing of the sensors.^23^

### 4.1. Fourier transform infrared spectroscopy (FTIR)

**Fig. 1:**
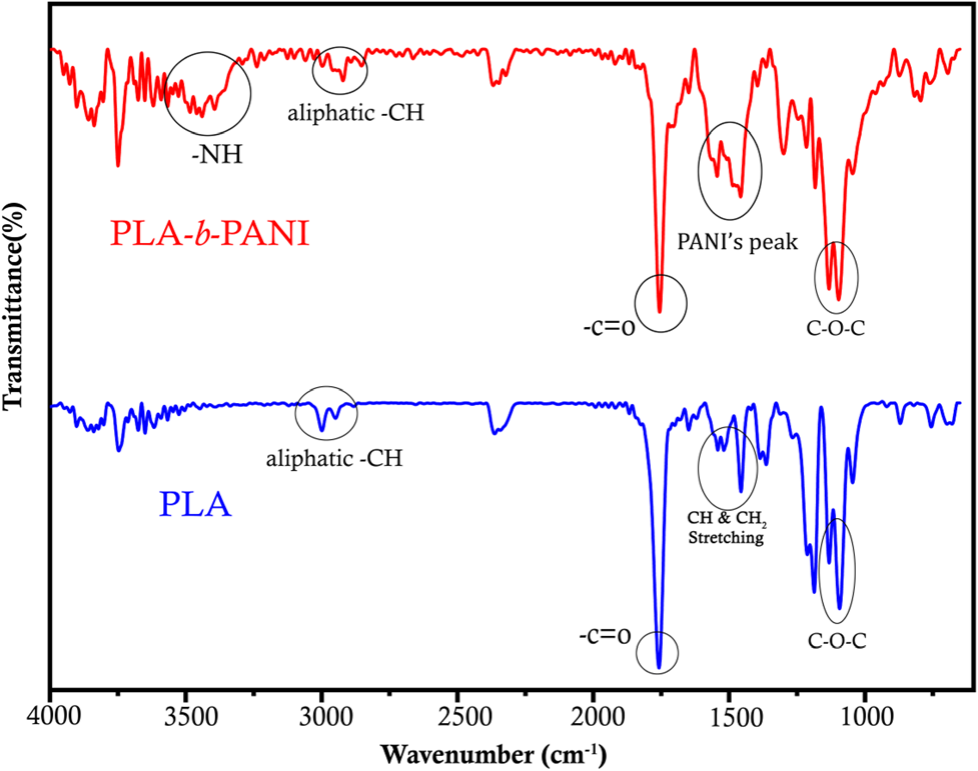
FT-IR spectra of PLA and PLA-*b*-PANI.

FT-IR spectra of PLA and the PLA-*b*-PANI are shown in Fig. 1. The spectrum of PLA shows characteristic peaks at around 1100, 1450 and 1750 cm^−1^, which are attributed to vibrational bands of C–O–C, C–H bonds and C=O stretching of esteric groups on PLA chains, respectively. In addition, peaks around 2980–3000 cm^−1^ indicate the stretching vibrations of aliphatic C–H bonds. Also, in the red spectrum, displayed peaks at around 1438 and 1556 cm^−1^ corresponding to the C=C stretching of the quinoid ring, and the C=C stretching vibration of the benzenoid ring, respectively. The appearance a peak at around 3400 cm^−1^ prove the vibrations of N–H in polyaniline groups demonstrates the formation of PANI segment in final polymeric structure.^24,25^

### 4.2. NMR spectroscopy

**Fig. 2:**
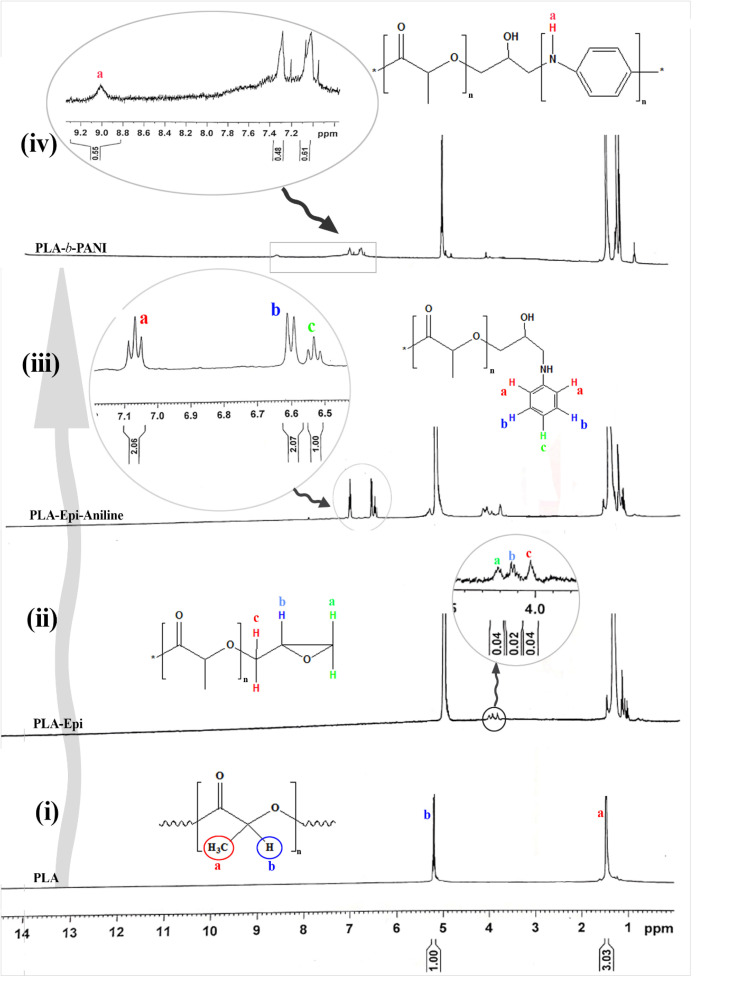
NMR spectra of PLA (i), PLA-Epi (ii), PLA-Epi-aniline (iii), PLA-*b*-PANI (iv).

The NMR spectra of the produced intermediate at each step of synthesis are given in Fig. 2. The spectrum of PLA is shown in Fig. 2(i). The corresponding chemical shifts at 1.5 and 5.2 ppm is referred to the two types of hydrogen in the PLA structure.^26^ Furthermore, for the sample of PLA-Epi, three new chemical shifts in the range of 4 to 4.3 ppm (Fig. 2(ii)) are related to the hydrogens in the epichlorohydrin moiety which proves the attachment of epichlorohydrin to the ends of PLA.^27^ The peaks observed at 6.5 and 7.1 ppm in Fig. 2(iii) relate to the aniline ring in polymeric structure. Finally, in the spectrum of ultimate synthesized copolymer (Fig. 2(iv)), the characteristic peak at 9.2 ppm is ascribed to the exchange proton of NH groups of aniline, which indicates the presence of PANI block in the copolymeric structure.^28^

### 4.3. GPC analysis

**Fig. 3:**
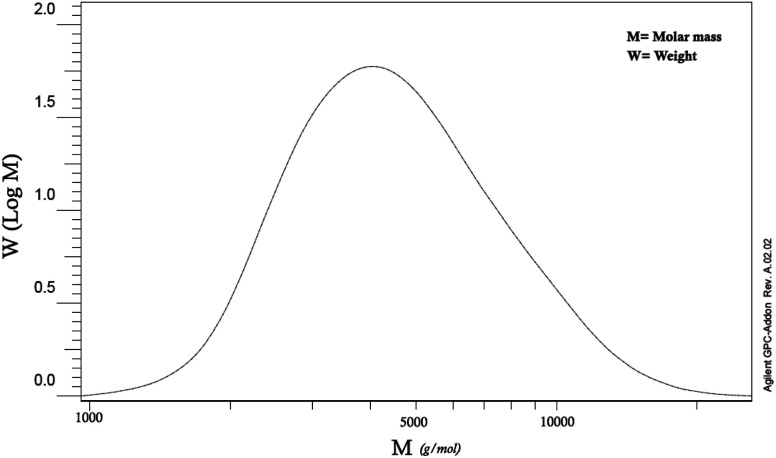
GPC diagram of PLA.

Based on the GPC test, the molecular mass of the synthesized PLA was indicated to be around 5000 g mol^−1^ (Fig. 3). Since, in the direct synthetic method of poly-esterification due to the presence of water in the reaction medium, the reversible equilibrium happens and makes it difficult to reach a high molar mass for synthesized polymers.^16^ However, by removing more water from the reaction medium under reduced pressure, the slightly higher molecular mass can be obtained. Moreover, the molecular weight of the copolymer was estimated based on ^1^H-NMR data as reported in previous ref. 29 and using the integration of epichlorohydrin peaks from Fig. 2(iv). The molecular weights are equivalent to 6371 and 1242 kDa for polylactic acid and polyaniline segment, respectively.

### 4.4. Thermal analysis

#### 4.4.1. Thermogravimetric analysis (TGA)

**Fig. 4:**
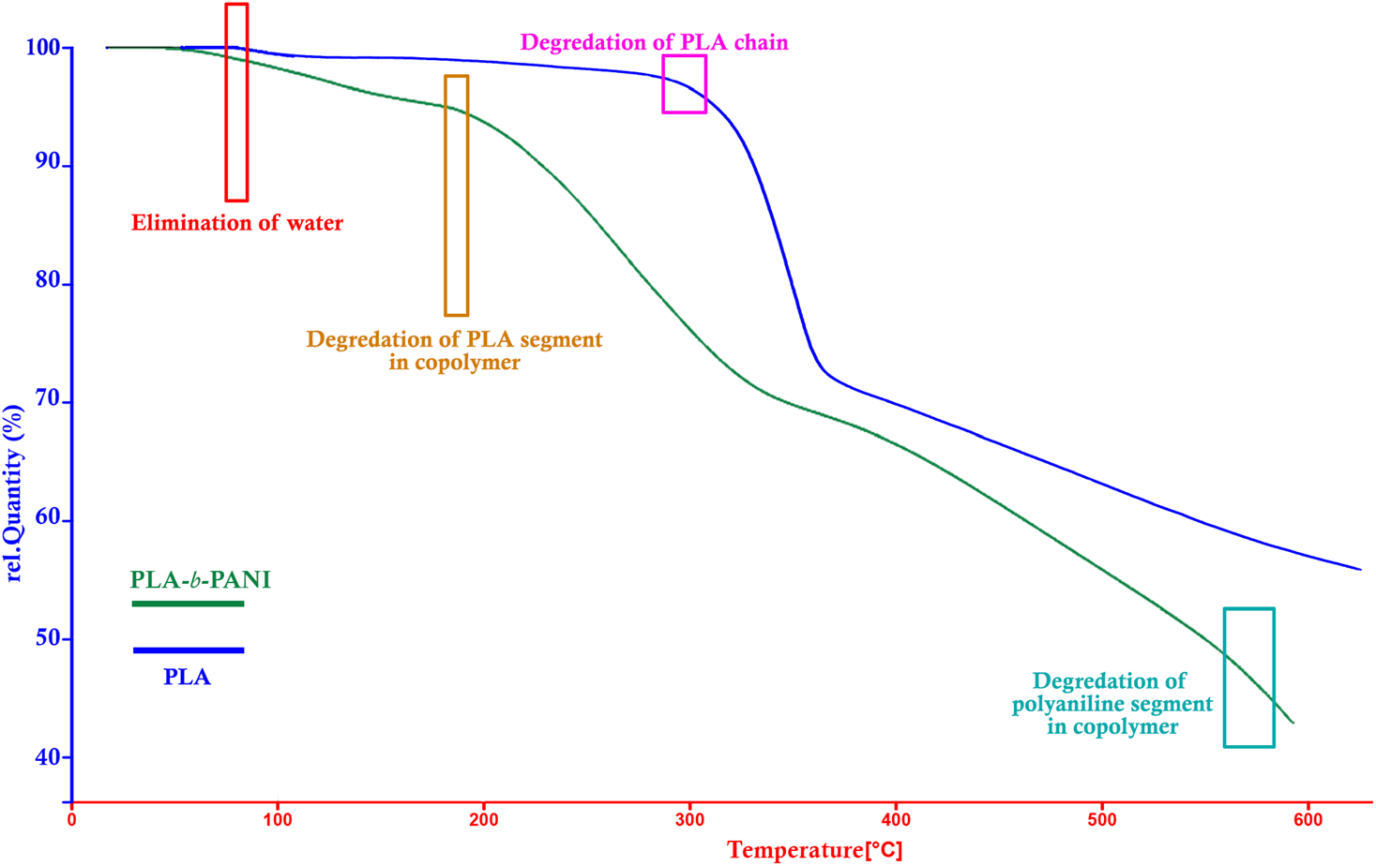
TGA diagram of PLA & PLA-*b*-PANI.

Fig. 4 shows the TGA thermograms of PLA and PLA-*b*-PANI obtained under N_2_ atmosphere at room temperature up to 600 °C. In the blue curve for PLA homopolymer, the weight loss (about 2%) at around 100 °C referred to elimination of water and peak in 350 °C referred to the degradation of main chain of PLA,^30^ and residue char is 60% at around 600 °C.

By comparing TGA thermograms of PLA-*b*-PANI (green curve) with PLA (blue curve) in Fig. 4, three degradation stages can be observed for PLA-*b*-PANI sample. The first stage before 200 °C is corresponding to removal of water and other volatile decomposition, and the second stage in the temperature range of 180 to 200 °C is caused by elimination of carboxyl group of PLA segment in copolymer. The third weight loss at around 550 to 600 °C referred to the polyaniline chain decomposition. It is found that due to the presence of HCl dopants in copolymer structure, degradation of estric group in polylactic acid chains is more straightforward, and started in lower temperature.^31^ In addition, the PLA-*b*-PANI chain is less thermally stable than the PLA homopolymer due to the presence of C–N bonds on the copolymer chains, which is less resistant than the C–C bonds. However, using the obtained results and the efficiency of copolymer char, it can be concluded that the obtained copolymer has good thermal stability and can be used as a reliable compound in sensor application.

#### 4.4.2. Differential scanning calorimetry (DSC)

**Fig. 5:**
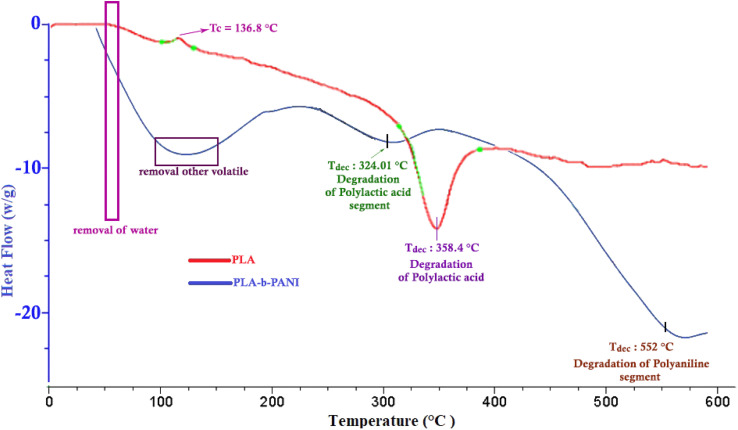
DSC diagram of PLA & PLA-*b*-PANI copolymer.

DSC analysis for PLA (red diagram) and PLA-*b*-PANI (blue diagram) are given in Fig. 5. The DSC thermogram of PLA shows two peaks. The first endothermic peak was attributed to cold crystallization of PLA at 136.8 °C and the second exothermic peak was regarded to decomposition of the PLA. The DSC thermogram of copolymer (the blue one) shows three peaks. The first peak is related to the removal of water and other solvents. The second peak at 324 °C same as the second peak in PLA diagram is regarded to decomposition of PLA part in copolymer. Eventually, the peak at 552 °C is attributed to decomposition of PANI segment in copolymer. According to the DSC and TGA diagrams, it was proved that the thermal stability of the copolymer is lower than that of the homopolymer when the PLA homopolymer is converted to PLA-*b*-PANI. In addition, the crystallinity of PLA in the copolymer is reduced.^32,33^

### 4.5. Scanning electron microscopy (SEM)

**Fig. 6:**
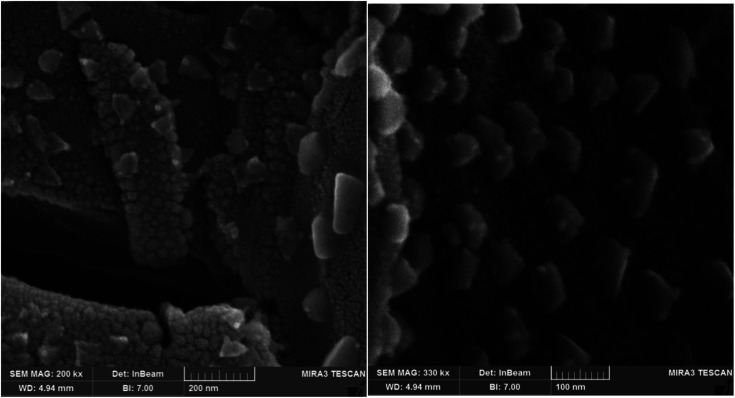
Structure of the synthesized PLA-*b*-PANI copolymer.

The nanostructure of the synthesized PLA-*b*-PANI is shown in Fig. 6. The morphology of the nanoparticles was evaluated by FE-SEM in two magnifications. The SEM images revealed that the morphology of PLA-*b*-PANI is like nano-spherical particles with a diameter of less than 100 nm.

### 4.6. Conductivity

The electrical conductivity of PLA-*b*-PANI was measured by a four-probe technique. For this purpose, the enough amount of copolymer (0.3 g) was prepared in a tablet shape (diameter: 13 mm, thickness: 1 mm) under a pressure of 14 MPa. The conductivity was calculated by the subsequent equation:
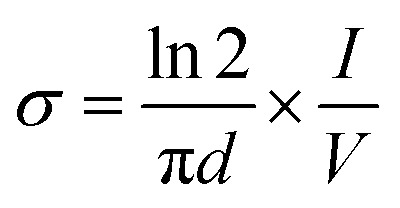
That *σ* is electrical conductivity, *V* is the voltage measured across inner probes, (*I*) is the applied current in the outer probes, and (*d*) is the tablet thickness (cm). The measured electrical conductivity of the PLA-*b*-PAN was 6.5 S cm^−1^. The conductivity of copolymer is lower than the polyaniline homopolymer. It was assumed that the decrease in the electrical conductivity of the copolymer is due to the presence of the polylactic acid with polyesters group in the PLA-*b*-PANI structure, which it acts as insulation for charge transfer through copolymer chains.^21^

### 4.7. Electrochemical characterization

EIS as a potent analytical tool was used for analyzing the complex electrochemical properties of a system, and is very sensitive to investigate the interaction between biomolecules and substrate. Impedance spectra can be analyzed by fitting to an equivalent electrical circuit to obtain the values of the electrical components that model each interfacial phenomenon, thus permitting the illumination of the processes of charge transfer and charge separation.^34,35^ The Randles equivalent circuit is widely used to simulate EIS data. This circuit incorporates both the resistance of electrolyte solution (*R*_s_), the double layer capacitance (*C*_dl_) generated by ions accumulating at electrode surfaces. Moreover, by performing the faradaic reaction on the surface of the electrode, two other parameters including resistance to charge transfer by faradaic reactions (*R*_ct_) and Warburg diffusion resistance (*W*) parallel to the double layer charge (*C*_dl_) are observed.^36^ A CV and EIS response was examined for characterization of the electrochemical profiles of PLA-*b*-PANI immersed in a solution containing Fe(CN)_6_^3−/4−^ (5 mM) and KCl (0.1 M).

**Fig. 7:**
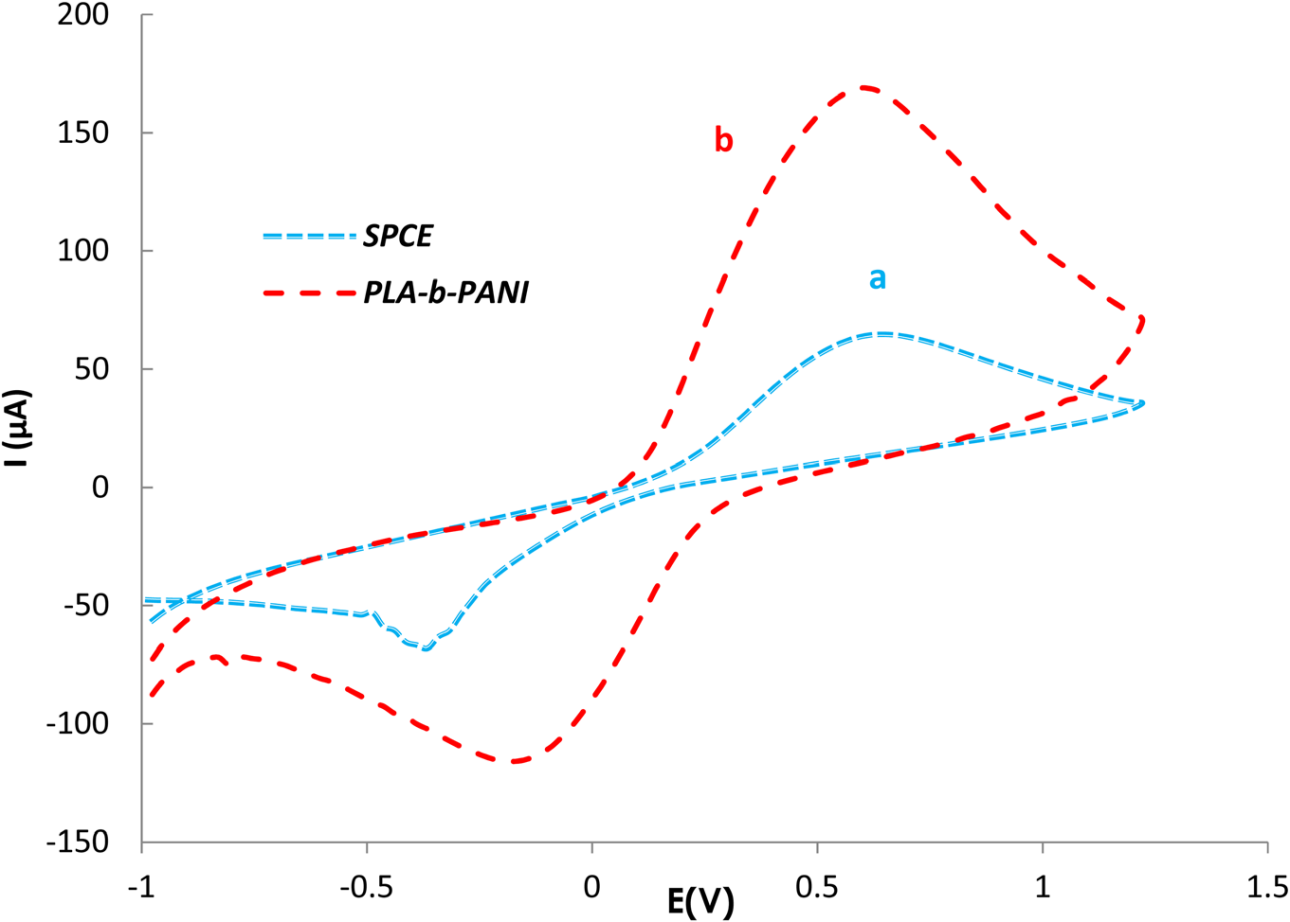
CVs of SPCE (a) and PLA-*b*-PANI/SPCE (b).

The CVs of SPCE (a) and PLA-*b*-PANI/SPCE (b) are shown in Fig. 7. After electrode modification with PLA-*b*-PANI, the current response of the Fe (CN)_6_^3−/4−^ increased obviously. This indicated that the PLA-*b*-PANI layer ameliorated the electrocatalytic activity of the electrode. The electrochemical properties of the coating also enhanced the electron transfer rate and promoted the redox reaction.^37^
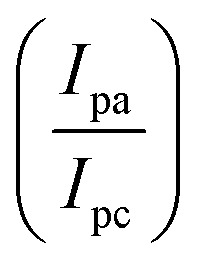
 is an indication of the rate of electrochemical reactions occurring at the surface of the electrodes. It also provides information about the diffusion coefficient of the species involved in the reaction. The ratio can be used to compare different systems and materials to identify the most efficient ones. The best reversibility gives the ratio as unity. The PLA-*b*-PANI/SPCE showed excellent reversibility 
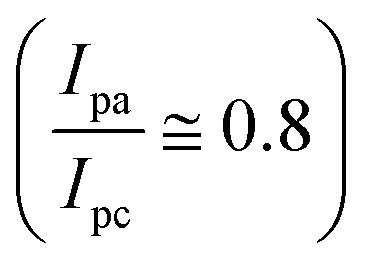
.^38^

**Fig. 8:**
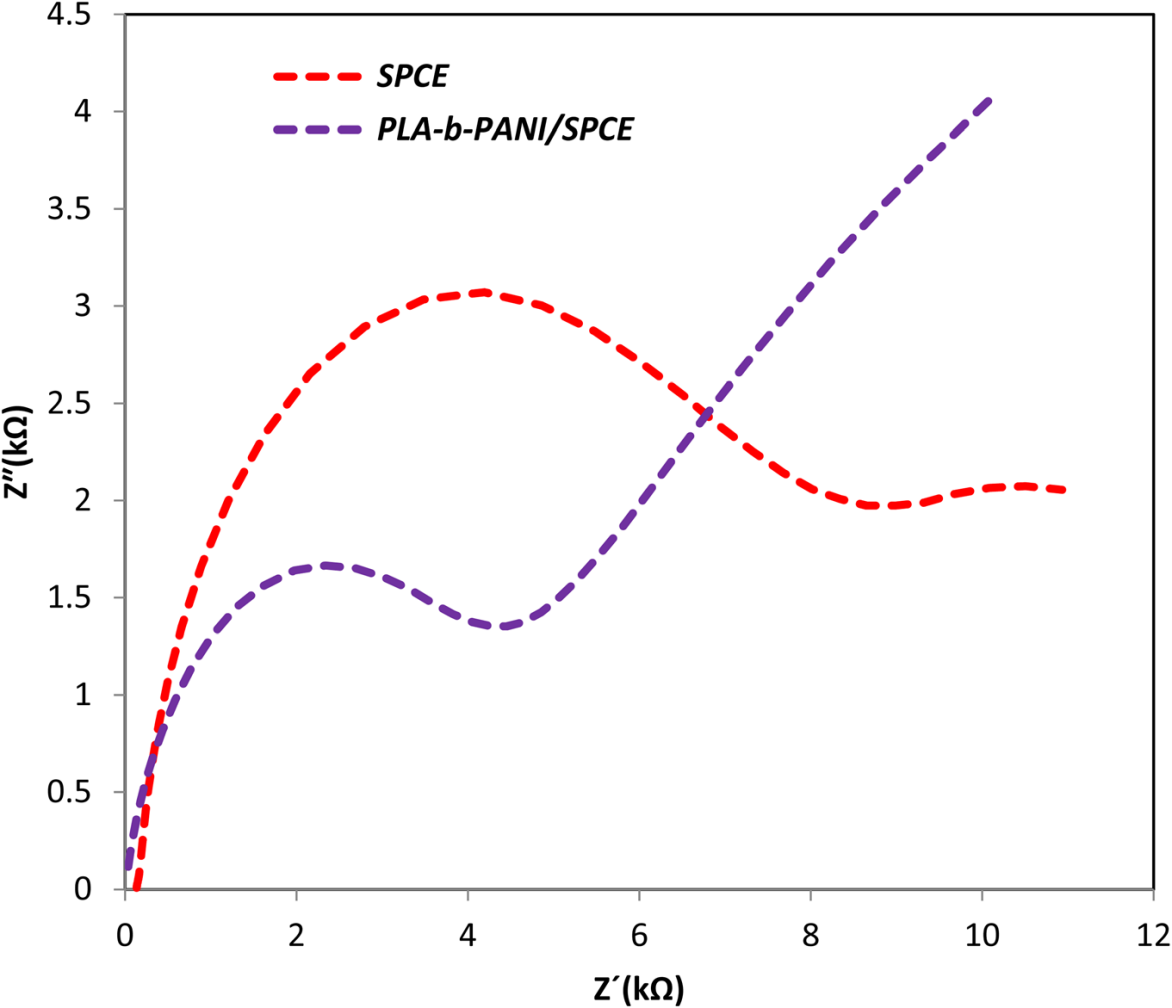
Displays EIS of SPCE and PLA-*b*-PANI/SPCE in Fe(CN)_6_^3−/4−^ (10 mM) and KCl (0.1 M).

EIS was carried out in the frequency range of 0.1 to 100 MHZ, to investigate the capacitive behavior and charge transfer mechanism, which involved in the performance of SPCE and PLA-*b*-PANI/SPCE between the electrolyte and electrode. As shown in Fig. 8, there was a semicircle at higher frequencies, which indicates the charge transfer resistance between the electrode and the electrolyte. This phenomenon depends on the diffusion of electrolyte ions to the electrode interface. Moreover, at lower frequency, there is a straight line, indicating the capacitive nature of the material. The radius of the semicircle is directly proportional to *R*_ct_ value. The *R*_ct_ values of SPCE and PLA-*b*-PANI/SPCE was 6.55 and 4.36 kΩ respectively. The results demonstrated that *R*_ct_ amount decrease when conductive PLA-*b*-PANI was added over the SPCE.^39^

### 4.8. EIS measurement

Albumin adsorption phenomenon involves the interaction of albumin and PLA-*b*-PANI surface without chemical change of albumin structure. In fact, the electrostatic interaction between the positive charges of PANI segment in copolymer and negative charges of BSA leads to this adsorption and the formation of an albumin monolayer or a multilayer film of modified electrode with copolymer. Therefore, effective parameters such as pH, substrate amount and adsorption time values on adsorption of BSA onto PLA-*b*-PANI has been investigated.

#### 4.8.1. Optimization of pH

**Fig. 9:**
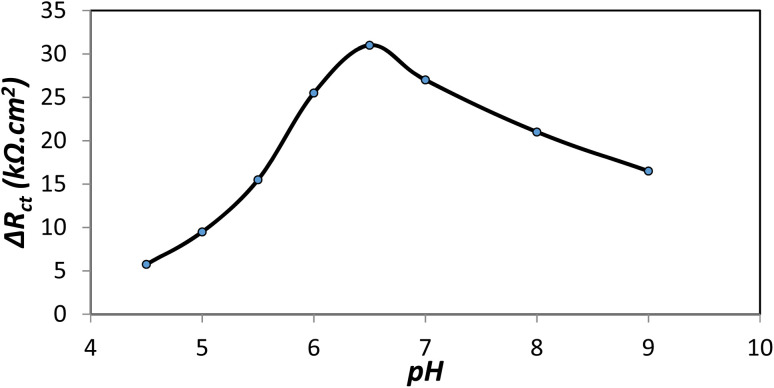
Effects of pH on impedimetric response of BSA (15 μg L^−1^).

Fig. 9 illustrates changes in Δ*R*_ct_ as a function of pH changes. When the pH amount changes from 4.5 to 6.5, the value of Δ*R*_ct_ increases. The isoelectric point of BSA is around pH 5. In the pH lower than 5, the BSA has positive charges and in the upper pH (greater than isoelectric pH), it has negative charges. Since, copolymer has more positive charges in the lower pHs (acidic condition), repulsion between BSA and surface is significantly high. By increasing the pH value higher than the isoelectric point, adsorption of BSA increases on the surface of PANI-*b*-PLA and the maximum amount is observed in pH 6.5.

The electrochemical data showed that the Δ*R*_ct_ values decrease at basic pH, where the protein is negatively charged and the substrate is neutral. The increasing in pH affords to the reorientation of the protein structure on the surface of the electrode and the formation of holes, in addition it causes to decrement in electrostatic attraction which lead to more effortless electron transfer.^40^

#### 4.8.2. Optimization of the PLA-*b*-PANI amount for fabrication of sensor

The effect of PLA-*b*-PANI drop coating amount on the modified electrode was investigated. The changes in *R*_ct_ values (Δ*R*_ct_) were studied for different drop coating values (0 to 12.5 μL with 2 mg mL^−1^ concentration). The thickness of the coating layer was determined based on the volume of the dispersion solution used for coating, which was adequate for the sensitivity of the detection method.^41^

**Fig. 10:**
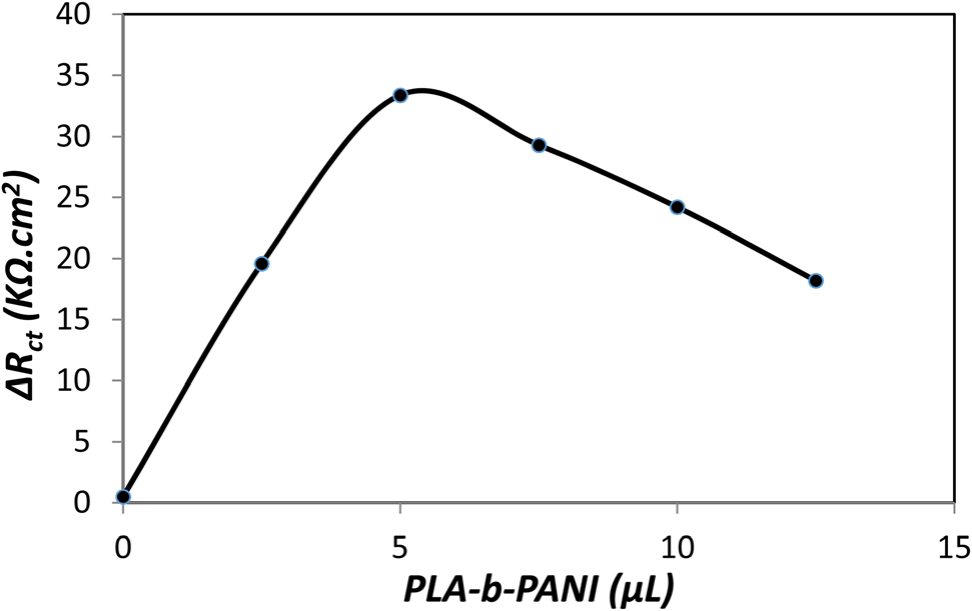
The effect of PLA-*b*-PANI amount on impedimetric response (BSA 15 μg L^−1^).

The result showed that coating a certain amount of PLA-*b*-PANI increased the conductivity and chemical sensitivity of the electrode. Fig. 10 proves that Δ*R*_ct_ increases with the addition in the volume of the drop coated copolymer. And also in the presence of 5 μL of PLA-*b*-PANI, it reaches a maximum and then decreases due to the increase of the active surface of the electrode. The decrease in Δ*R*_ct_ with the increase of PLA-*b*-PANI can be attributed to the increase in the contact resistance of the copolymer, which is caused by the increase in the coating layer thickness. As a result, a drop coating volume of 5 μL was selected for further analysis.^42^

#### 4.8.3. Optimization of incubation time

**Fig. 11:**
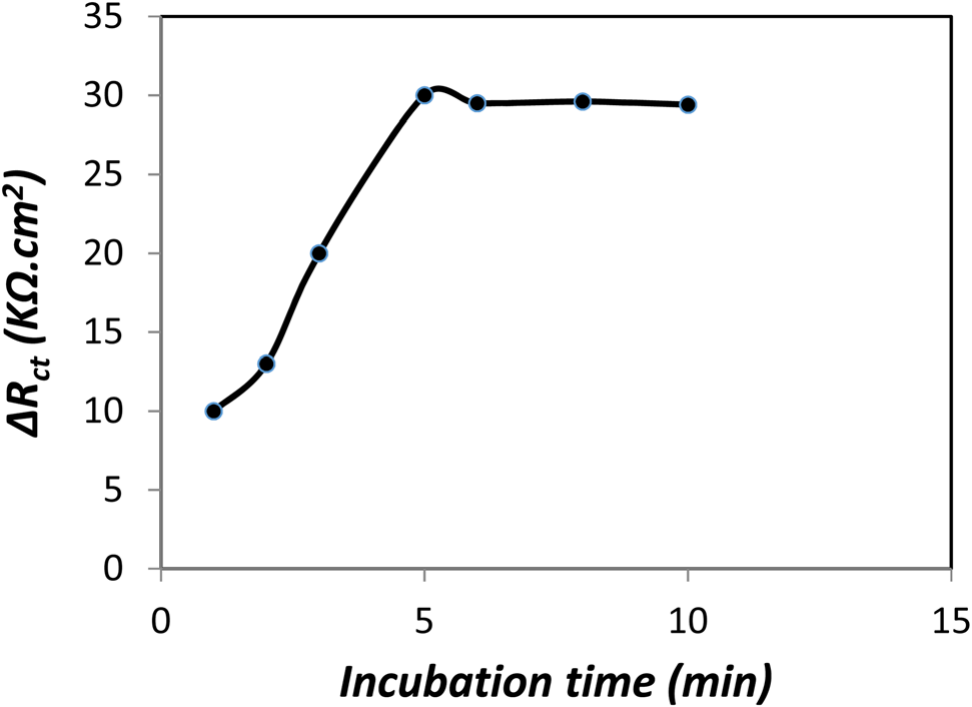
Effect of incubation time on impedimetric response (Δ*R*_ct_) in phosphate buffer solution (pH = 6.5).

The incubation time is a critical parameter that affects the adsorption of BSA on the surface of modified electrode and consequently, the sensitivity of the detection method. The binding time of BSA (10 μg L^−1^) to the electrode surface was studied in the range of 1–10 min. Impedimetric response change with incubation time is shown in Fig. 11. A significant change in Δ*R*_ct_ value was observed with increasing time up to 5 min and then plateaued with further increase in incubation time. Therefore, the incubation time of 5 min was chosen as the optimal time.^43^

#### 4.8.4. Analytical characteristics of BSA impedimetric sensor

**Fig. 12:**
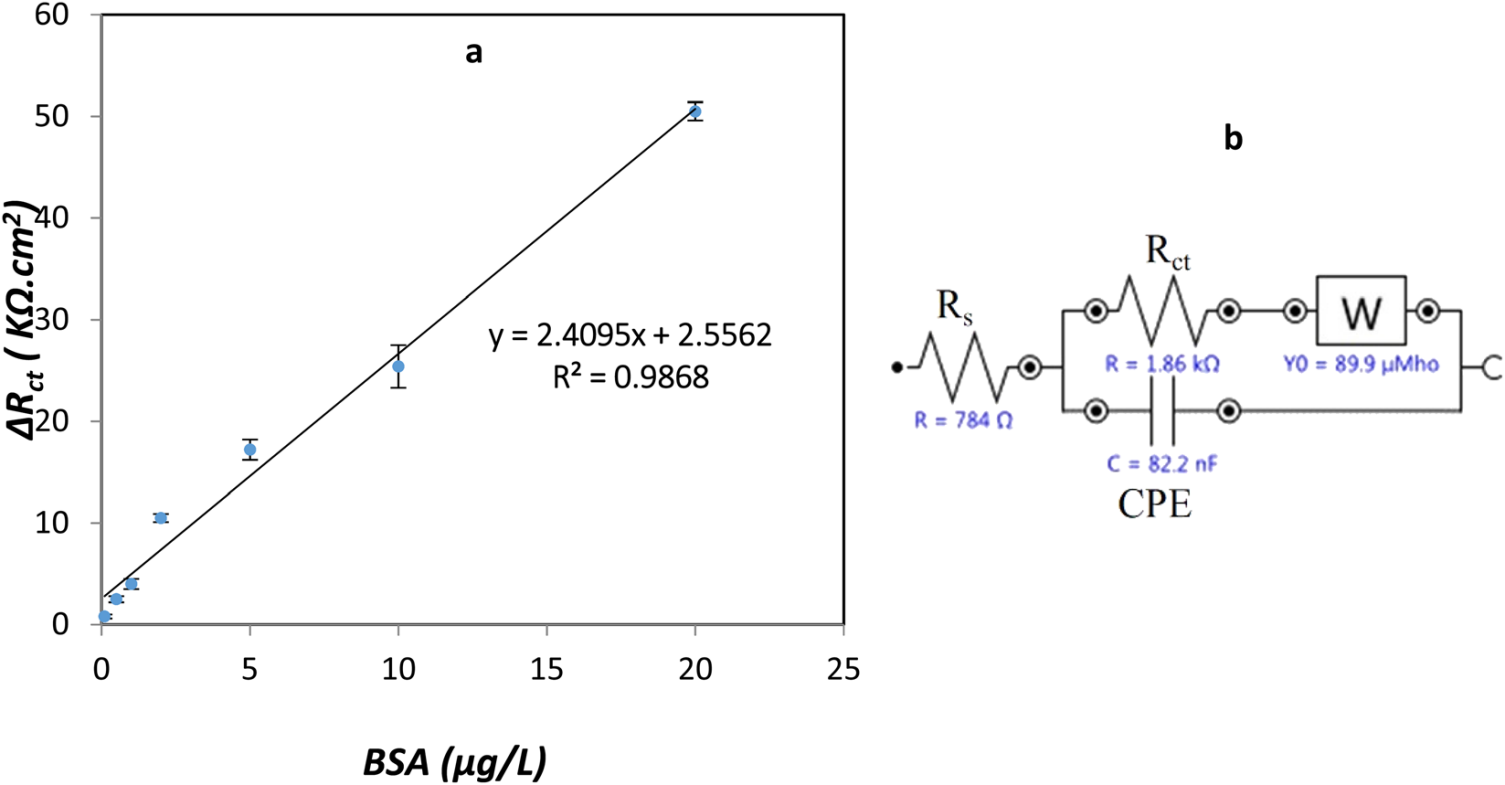
(a) Impedimetric response of PLA-*b*-PANI/SPCE with BSA concentration ranging from 0.10 to 20 μg mL^−1^ in PBS (0.1 M, pH 6.5), (b) equivalent circuit of PLA-*b*-PANI/SPCE of GCE.

For investigating the feasibility of measuring the BSA by the proposed sensor, the impedance spectrum of the modified electrode was recorded in the presence of [Fe(CN)_6_]^3−/4−^ as a redox probe to determine different amounts of BSA under optimal conditions. The response of the sensors can be analyzed by fitting an equivalent electrical circuit shown in Fig. 12b. Therefore, impedance measurements were performed to evaluate the electrode *R*_ct_ and the double-layer capacitor (CPE) behavior. Fig. 12a shows the calibration curve of the change in charge transfer resistance Δ*R*_ct_*versus* concentration of BSA.^44^

Based on the curve, with increasing in the BSA concentration in solution, the Δ*R*_ct_ value was increased. These observations can be related to this fact that BSA adsorbed on the electrode surface lead to decrease in electron transfer and increases in the amount of *R*_ct_. There is a linear relationship between Δ*R*_ct_ and BSA concentration from 0.1 to 20 μg L^−1^ and the correlation coefficients are more significant than 0.98.^20,35,45^

## 5. Conclusion

In the present work, the amphiphilic block copolymer of PLA and PANI (the PLA-*b*-PANI) was synthesized for the first time by using the sequential polymerization method, and was successfully used as a nano-substrate to interact with protein to make an impedimetric sensor. The final synthetic copolymer was characterized by several analyses. The copolymer nanostructure was confirmed by FE-SEM images. The molecular weight of homopolymer and copolymer was calculated by GPC and NMR. The PLA-*b*-PANI/SPCE electrode is used as an impedimetric sensor for the detection of BSA. In this regard, the influence of effective parameters such as changes in pH, amount of copolymer, BSA concentration and incubation time in the adsorption of BSA on the modified electrode was investigated. The outcomes showed promising results in repeatability, reproducibility, and stability within the linear concentration range of 0.1–20 μg L^−1^ of BSA, with a detection limit of 0.05 μg L^−1^. All the findings demonstrate that the final synthetic copolymer has potential activity for use as an efficient modified electrode in fabrication of the reproducible and flexible biosensor in lab-on-chip technology. So, PLA-*b*-PANI copolymer is a sensitive substrate for BSA adsorption that can feasibly be applied in protein Impedimetric sensors.

It is expected that the newly introduced copolymer can be used in the development of electrochemical biosensors and used for recovery biomolecules from biological environments and food waste.

## Abbreviation

BSA: bovine serum albumin
PLA: polylactic acid
PANI: polyaniline
PLA-*b*-PANI: polylactic acid-*block*-polyaniline copolymer
EIS: impedance spectroscopy
CV: cyclic voltammetry
FT-IR: Fourier transform infrared spectroscopy
DSC: differential scanning calorimetric
TGA: thermogravimetric analysis
SEM: scanning electron microscopy
CPs: conductive polymers

## Conflicts of interest

There are no conflicts to declare.

## Supplementary Material
